# Research on segmentation model of optic disc and optic cup in fundus

**DOI:** 10.1186/s12886-024-03532-4

**Published:** 2024-06-28

**Authors:** Naigong Chen, Xiujuan Lv

**Affiliations:** 1 National Clinical Research Center for Ocular Diseases, Eye Hospital, Wenzhou Medical University, Wenzhou, 325000 China; 2 State Key Laboratory of Ophthalmology, Optometry and Vision Science, Eye Hospital, Wenzhou Medical University, Wenzhou, 325027 China; 3 National Engineering Research Center of Ophthalmology and Optometry, Eye Hospital, Wenzhou Medical University, Wenzhou, 325027 China

**Keywords:** Glaucoma screening, YOLO model, Deep learning, Fundus image segmentation, REFUGE dataset

## Abstract

**Background:**

Glaucoma is a worldwide eye disease that can cause irreversible vision loss. Early detection of glaucoma is important to reduce vision loss, and retinal fundus image examination is one of the most commonly used solutions for glaucoma diagnosis due to its low cost. Clinically, the cup-disc ratio of fundus images is an important indicator for glaucoma diagnosis. In recent years, there have been an increasing number of algorithms for segmentation and recognition of the optic disc (OD) and optic cup (OC), but these algorithms generally have poor universality, segmentation performance, and segmentation accuracy.

**Methods:**

By improving the YOLOv8 algorithm for segmentation of OD and OC. Firstly, a set of algorithms was designed to adapt the REFUGE dataset’s result images to the input format of the YOLOv8 algorithm. Secondly, in order to improve segmentation performance, the network structure of YOLOv8 was improved, including adding a ROI (Region of Interest) module, modifying the bounding box regression loss function from CIOU to Focal-EIoU. Finally, by training and testing the REFUGE dataset, the improved YOLOv8 algorithm was evaluated.

**Results:**

The experimental results show that the improved YOLOv8 algorithm achieves good segmentation performance on the REFUGE dataset. In the OD and OC segmentation tests, the F1 score is 0.999.

**Conclusions:**

We improved the YOLOv8 algorithm and applied the improved model to the segmentation task of OD and OC in fundus images. The results show that our improved model is far superior to the mainstream U-Net model in terms of training speed, segmentation performance, and segmentation accuracy.

## Background

Glaucoma is a chronic progressive optic neuropathy and one of the leading causes of irreversible blindness in the world [1]. According to the World Health Organization, about 80 million people worldwide suffer from this disease [2], and it is expected to increase to about 111.8 million people by 2040 [3]. The loss of vision caused by glaucoma is due to elevated intraocular pressure in the optic nerve, it is usually asymptomatic. Therefore, early diagnosis of glaucoma is crucial to prevent irreversible vision loss.

**Fig. 1:**
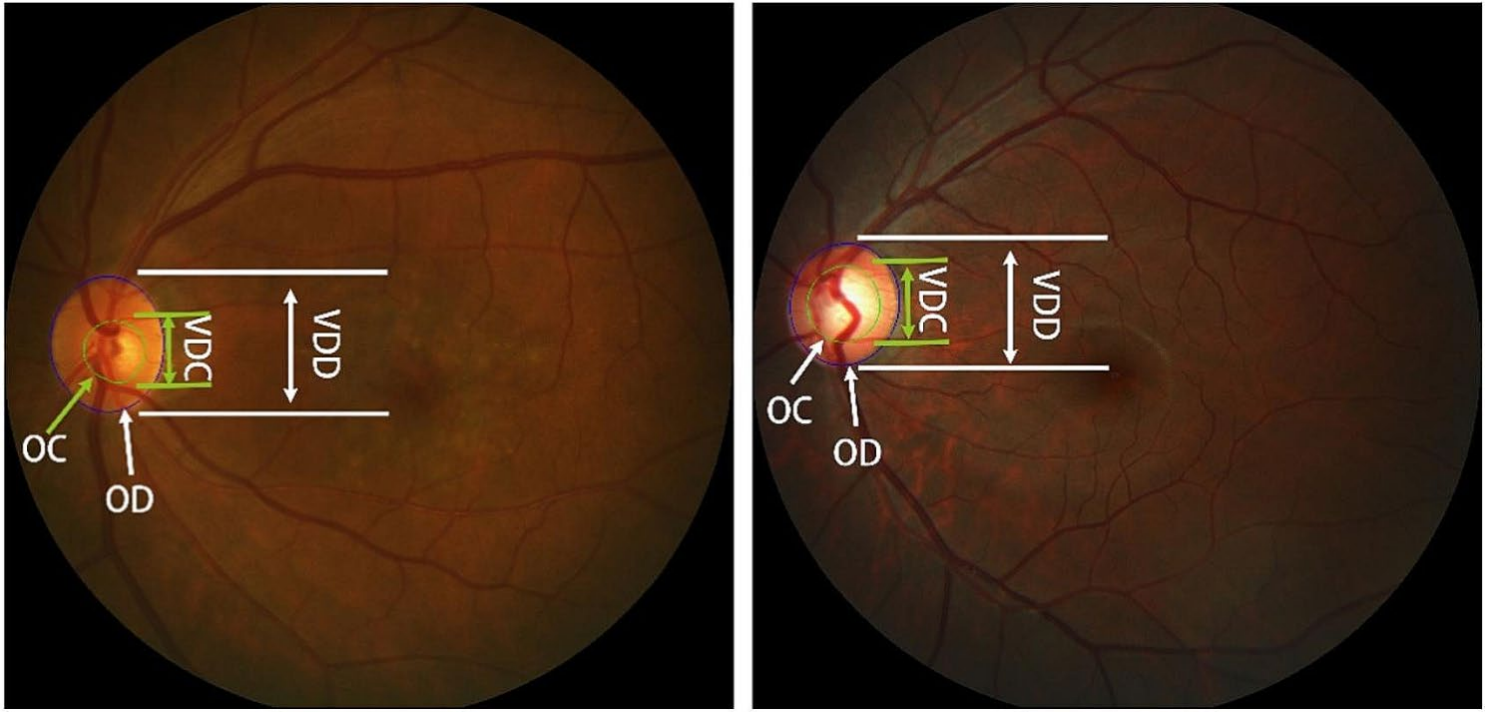
Marking of optic disc and cup (the original image is from REFUGE dataset). (VDC: vertical diameter of the optic cup, VDD: vertical diameter of the optic disc, OC: optic cup, OD: optic disc)

Currently, common diagnostic methods for glaucoma include intraocular pressure assessment [4], optic nerve head (ONH) assessment [5], and functional perimetry [6]. Functional perimetry measures the range of vision when the patient’s line of sight is focused on the center point. Due to the uneven equipment levels of various hospitals, not every hospital has perimetry instruments, so this examination cannot be widely used. Intraocular pressure assessment is usually measured with a tonometer, but high intraocular pressure is usually not a direct diagnosis of glaucoma. Therefore, considering the cost, in clinical practice, ophthalmologists usually manually measure the vertical cup-disc ratio (vCDR) [7]of fundus images to assess the ONH. It is generally believed that a vCDR value greater than 0.5 indicates a higher risk of glaucoma [8, 9]. Figure 1 (left) shows a normal fundus image and vCDR related annotations, and Fig. 1 (right) shows a glaucoma fundus image and vCDR related annotations.

However, manual assessment of ONH consumption consumes a significant amount of human labor and is not suitable for large-scale screening. Moreover, manual assessment relies heavily on the experience of clinicians, and skilled doctors require approximately 8 min to completely separate the OD and OC of one eye [10]. Therefore, a computer-assisted model that can accurately segment the OD and OC is very important and valuable for large-scale screening of glaucoma, especially in medical institutions lacking sufficient professional doctors [11].

In recent years, with the improvement of computer computing power, many algorithms have been developed for segmenting OD and OC. They are mainly divided into traditional algorithms and deep learning algorithms.

### Traditional algorithm

Traditional algorithms are mainly divided into template matching-based algorithms and deformable model algorithms. The template matching-based algorithm mainly combines the prior shape information of the target to match the OD and OC boundaries as a circle or ellipse. The algorithm proposed by Roychowdhury et al., [12] first extracts the bright region near the blood vessels from the fundus image using morphology, then extracts the final OD from the bright region using Gaussian mixture model, and finally uses ellipses for fitting processing. Lalonde et al., [13] proposed a hausdorff-based template matching OD segmentation method, which uses pyramid decomposition and confidence assignment to locate the OD. Zheng et al., [14]combined prior information with OD and OC, using a function based on graph cutting technology to segment OD and OC. Some algorithms [15, 16] use circular or elliptical Hough transform, and after multiple image processing such as edge detection and threshold segmentation, fit the OD. The above template matching-based algorithms not only require good blood vessel detection algorithms, but also require a large number of sampling points, and may not be able to detect irregular edges of the object due to changes in the shape of the detected target.

The deformable model algorithm mainly initializes an initial OD or OC contour, and then deforms it towards the target contour by minimizing various energy terms. Energy terms are usually defined by image gradients, image intensity, and boundary smoothness [17]. Haleem et al., [18] proposed an adaptive edge smoothing update model (ARESM) that iteratively updates the contour by minimizing the energy function. Joshi et al., [19]proposed an improved Chan-Vese active contour model for OD segmentation, mainly by analyzing two texture feature spaces and local red channels near the pixel. Xu et al., [20] proposed an OD and OC segmentation algorithm based on snake model, which marks contour points as positive or non-positive after each deformation, and extracts object boundaries before the next contour deformation. Although deformable models sometimes achieve relatively ideal results, they are highly dependent on initialization and are susceptible to pathological changes in the eyes.

Most traditional algorithms are designed to segment fundus images based on specific contrast or image quality. Once these characteristics change, traditional algorithms can exhibit extreme instability and poor robustness. Moreover, traditional algorithms treat segmentation OD and segmentation OC as two separate tasks during the segmentation process, ignoring their relationship.

### Deep learning algorithm

Deep learning is a machine learning technique based on artificial neural networks, in which convolutional neural networks (CNN) have gradually emerged in various computer vision tasks [21, 22].Compared to traditional algorithms, deep learning-based algorithms can achieve better performance and versatility in an end-to-end manner. In the field of image segmentation, many segmentation models based on convolutional networks have been proposed, mainly including fully convolutional networks (FCN) [23], U-Net [24], and generative adversarial networks (GAN) [25].

For the fully convolutional network model, Mohan et al., [26] proposed a CNN network segmentation model named Fine-Net, which uses the feature extraction model of the full-resolution residual network. Subsequently, in order to improve the segmentation accuracy of OD, a model named P-Net was introduced as a prior network, which was cascaded with the Fine-Net model to generate high-resolution feature maps [27]. Liu et al., [28] proposed an end-to-end spatial perception neural network to segment OD and OC. First, CNN was used to extract spatial features, then a pyramid filtering module was used to obtain multi-scale features of the control, and finally the features were passed to the segmentation module to obtain the prediction results. Arous convolution was used in the proposed model architectures, as it can accurately adjust the receptive field of the network and obtain richer features in image segmentation tasks.

The U-Net model and its variants benefit from the U-shaped structure and skip connections, and have shown excellent performance on small datasets and are widely used in medical image segmentation tasks such as OD and OC segmentation. The U-Net model is an improved version based on the FCN model, and is more suitable for semantic simple and structurally fixed medical image segmentation. Since Ronneberger et al., proposed the U-Net model for medical image segmentation, many improved versions based on the U-Net model have been proposed for OD and OC segmentation. Fu et al., [29] proposed a multi-scale U-Net convolutional network named M-Net and polar coordinates to segment OD and OC. This Model uses four different sizes of regions of interest (ROI) as inputs to produce four outputs, and finally estimates the four outputs to obtain the results. Gu et al., [30] proposed a context encoder network named CE-Net for 2D medical image segmentation to segment OD and OC. Compared to M-Net, CE-Net performs better in the OD segmentation task. Yu et al., [31] improved U-Net by using the ResNet34 network architecture to segment OD and OC. The improved version uses two U-Net models, one for the extraction of the region of interest in the fundus image, and the other for the segmentation of OD and OC in the region of interest. Zhang et al., [32] proposed a transferable attention U-Net model that uses two discriminators and attention modules to extract invariant features from fundus images, thereby improving the generalization ability of the model. Although the above improved U-Net model allows features to be passed from the encoder to the decoder to preserve some spatial features to improve network performance, there may be feature differences between the two sets of features, and some features may be lost during the transfer process.

GAN models have developed rapidly in the field of computer vision in recent years. GAN models mainly consist of two modules: generator and discriminator. The generator constantly optimizes the data it generates so that the discriminator cannot distinguish it, and the discriminator also optimizes itself to make its judgment more accurate. The relationship between the two forms a confrontation, hence the name generative adversarial network. Wang et al., [33] proposed a patch-based output space adversarial learning framework (POSAL) to jointly segment OD and OC. In the following work, in order to improve the performance and accuracy of segmentation, they proposed a boundary-free and entropy-driven adversarial learning (BEAL) model [34].

In summary, traditional algorithms and deep learning algorithms can accurately segment the OD and OC of fundus images, but the performance and generality of deep learning algorithms are better. As long as the data during training is comprehensive, the final segmentation results are almost unaffected by image contrast and quality differences. However, these deep learning algorithms have problems such as long training and prediction time due to deep network structure or multi-stage feature acquisition, and inaccurate segmentation results caused by excessive feature loss. Moreover, these algorithms are classified from the perspective of image processing and classification, belonging to image semantic segmentation, which simply assigns each pixel in the image to its corresponding semantic category. It mainly targets pixels and is a pixel-level image segmentation method that cannot directly label objects. In recent years, with the development of object detection models, more and more research has combined object detection and semantic segmentation, and proposed the concept of instance segmentation. Instance segmentation divides each object in the image into independent instances, which can label the segmented instances. Among many instance segmentation models, the YOLO model (you only look once) has been widely applied due to its superior speed and performance. In this article, we mainly improve the v8 version of YOLO model for instance segmentation of fundus images OD and OC. The main improvements are as follows:

1. Directly obtain the region of interest (ROI) of the fundus image through traditional cutting-edge image algorithms.
2. Perform image enhancement processing on the acquired fundus image ROI to increase the amount of training data and prevent model underfitting.
3. Modify the loss function of YOLOv8 from the original CIoU to Focal-EIoU, which speeds up the convergence rate of fundus image training and improves the accuracy of the prediction results.

### Dataset processing

**Fig. 2:**
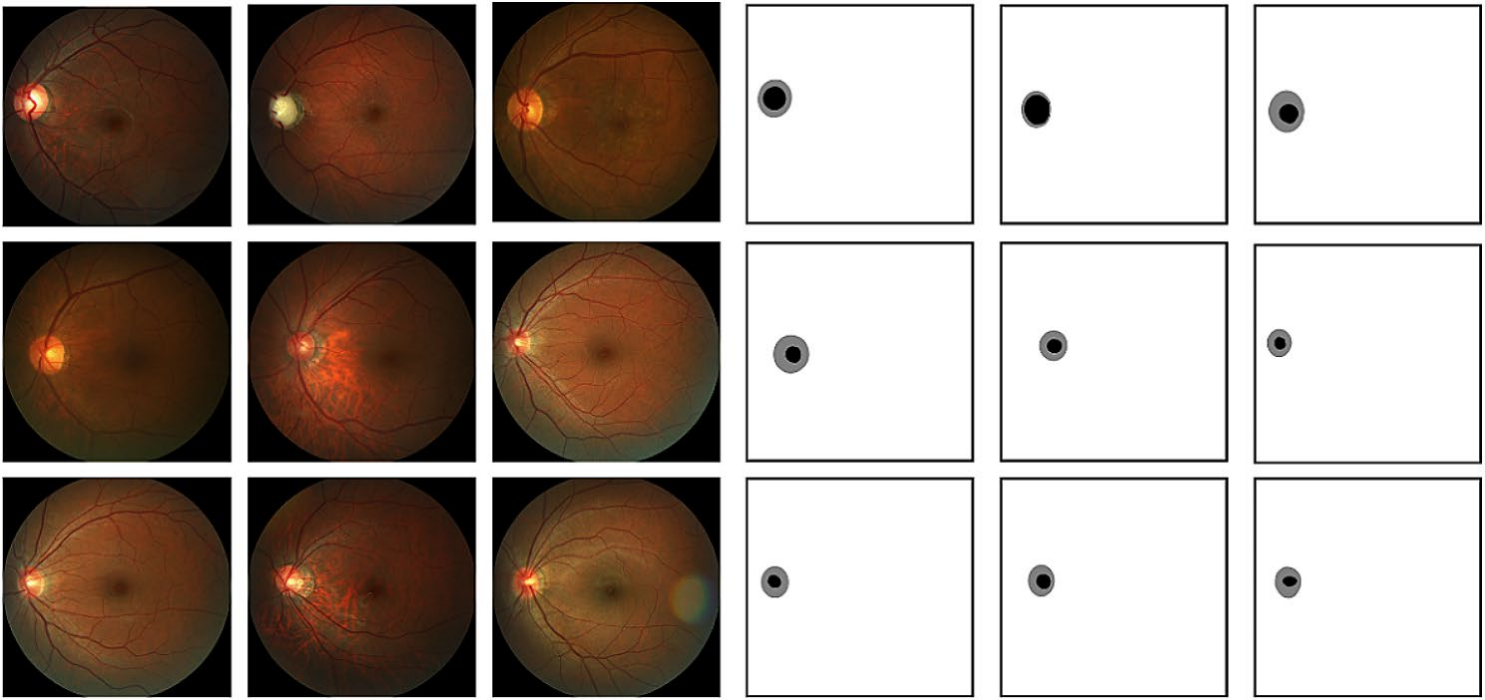
Marking of optic disc and cup (image from REFUGE dataset)

The training of the model in this article uses the REFUGE dataset [35], which consists of three sets of images: 400 training images, 400 validation images, and 400 test images. These images have two sizes: 2124 × 2056 pixels and 1634 × 1634 pixels. The training set consists of 40 images of the fundus of glaucoma and 360 images of the fundus without glaucoma. Each fundus image has annotations of the optic disc and optic cup, which are generated by 7 ophthalmologists with an average of 8 years of experience using majority voting [36]. Figure 2 (left) shows the original fundus image, and Fig. 2 (right) shows the annotations of the optic disc and optic cup.

### Preprocessing

The input of the YOLOv8 model contains the original image and multiple labeled coordinate values. Since the REFUGE labeling is a single image rather than multiple coordinate values, it is necessary to convert the REFUGE labeled image, extract the border, and adapt it to the input of the YOLOv8 model. Considering that the REFUGE labeled images are grayscale images, with a optic disc grayscale value of 128 and a optic cup grayscale value of 0, the extraction algorithm only needs to traverse the labeled image and extract based on the grayscale value. We named the extraction algorithm YR-Adapter. The main process of the YR-Adapter algorithm is as follows:

1. Traverse the pixel grayscale values of the label image from left to right and top to bottom.
2. The optic disc border is the leftmost and rightmost position with a gray value of 128 per row, and the optic cup border is the leftmost and rightmost position with a gray value of 0 per row.
3. If the amount of coordinate value data for the entire optic disc cup border is too large, it will affect the training speed. By actually counting the number of pixels in a frame, this paper ultimately determined that taking a coordinate every 15 pixels is more in line with the input data volume of the model. So this algorithm takes a coordinate for the border every 15 pixels.
4. Sort the coordinate points clockwise. Take the center point C of each coordinate, obtain each coordinate point P, calculate the orientation angle alphaP between the x-axis and the vector CP, and sort the point list according to the associated angle of the points.

**Fig. 3:**
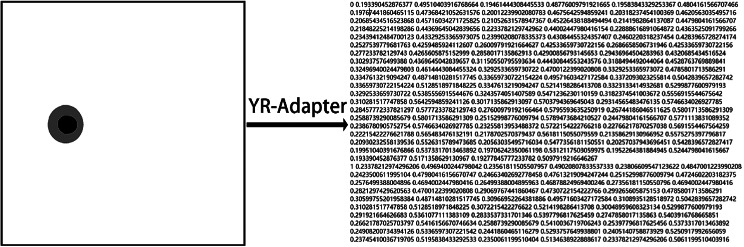
Processing result of YR-Adapter algorithm

Figure 3 shows the processing results of YR-Adapter.

### Enhancing processing

In order to enhance the fitting accuracy, the model in this article expands the training set through image enhancement techniques. The main enhancement techniques used are: Mosaic data enhancement [37], Mixup data enhancement [38], and LetterBox data enhancement.

The main idea of Mosaic enhancement technology is to randomly crop four images and then stitch them together into one image as training data. The steps are:

1. Randomly read four images from the dataset each time.
2. Perform operations such as flipping, scaling, and color gamut changes (changes to the brightness, saturation, and hue of the original image) on each of the four images. After the operation is complete, place the original image in the upper left, lower left, lower right, and upper right positions in the same manner as the first image.
3. Combine the images and frames. After arranging the four images, use a matrix to extract fixed areas from the four images and then stitch them together into a new image.

The Mixup enhancement technique is an algorithm that enhances images by mixing classes, allowing it to combine different images to expand the training dataset.

The LetterBox enhancement technique is relatively simple. It mainly scales the image to a specified size (scaling proportionally in height and width), and then adds black borders on both sides of the image to make it consistent with the size to be adjusted. This method can preserve the aspect ratio of the original image, while also making the image more suitable for input to image segmentation algorithms.

### Model network structure

**Fig. 4:**
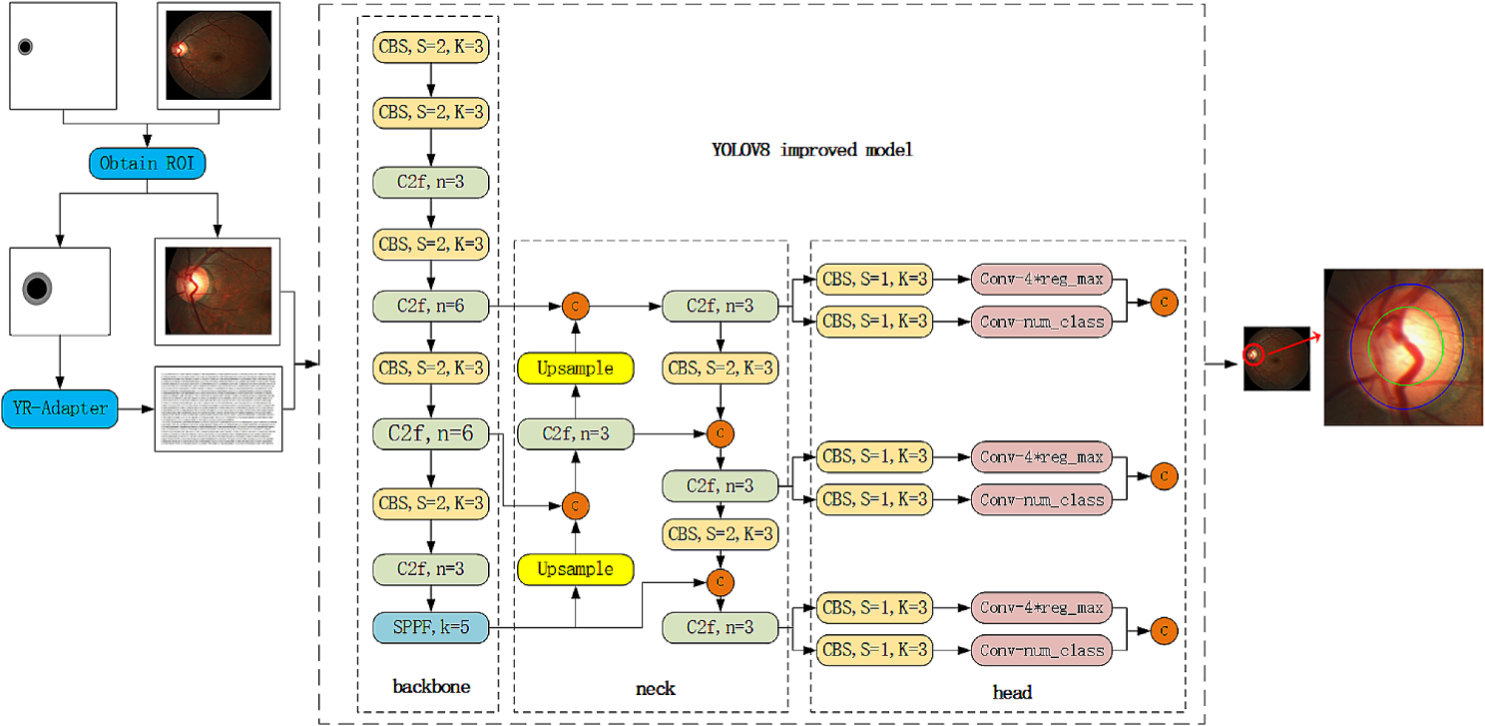
Improved model network structure

YOLO (You Only Look Once: Unified, Real-Time Object Detection) [39] was first proposed by Joseph Redmon and Ali Farhadi in 2015. In 2017, they proposed YOLOV2, and later YOLOV3. The latest version is YOLOV8, which is a standard one-stage object detection algorithm. Compared to Faster RCNN [40] and SSD [41], YOLO can better implement the idea of directly using regression methods to obtain the current target and target category problems that need to be detected. The core point of the YOLO algorithm is the input image, which uses a method of simultaneously predicting the location and category of multiple bounding boxes to detect the location and classification of the category. It is a more thorough end-to-end object detection and recognition method. Compared to Faster RCNN and SSD, it can achieve a faster detection speed. Figure 4 is a model architecture improved based on YOLOV8 in this article.

The YOLOv8 network model consists of three parts: backbone (main network), neck (feature enhancement network), and head (detection head).

- **backbone**: It is mainly used to extract feature information from fundus images for later network use.
- **neck**: between the backbone and the head, mainly to better utilize the features extracted by the backbone.
- **head**: Using the features extracted in the previous two sections, perform classification and regression to obtain categories and targets.

**Fig. 5:**
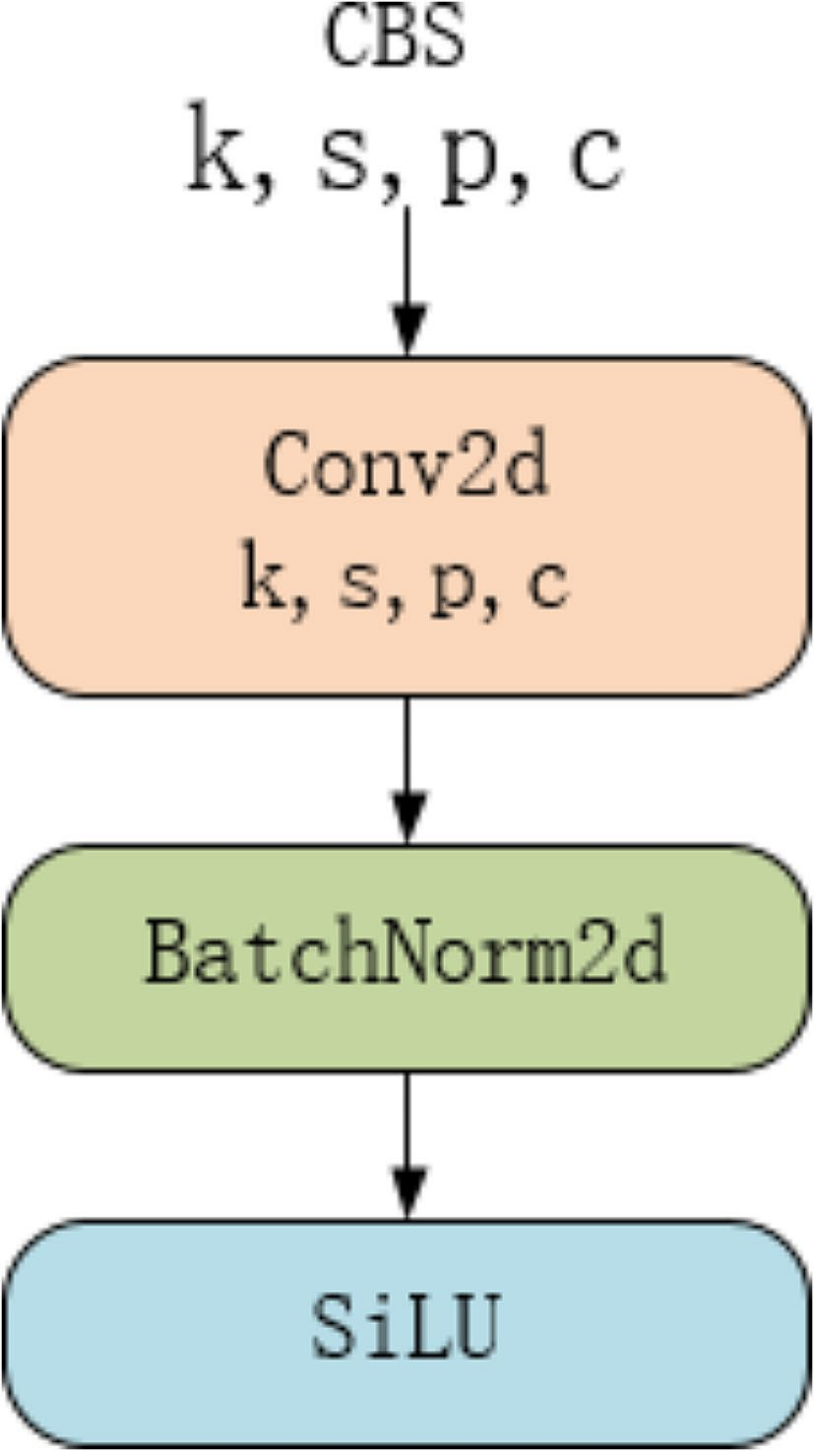
CBS architecture diagram

CBS consists of three parts: a 2D convolution, a 2D batch normalization, and a SiLU activation function, as shown in Fig. 5. Among them, Conv2d is a 2D convolution layer, where k represents the size of the convolution kernel, s is the stride, p is the padding mark, c represents the number of convolution kernels, BatchNorm2d is a normalization layer, and SiLU is an activation function layer.

**Fig. 6:**
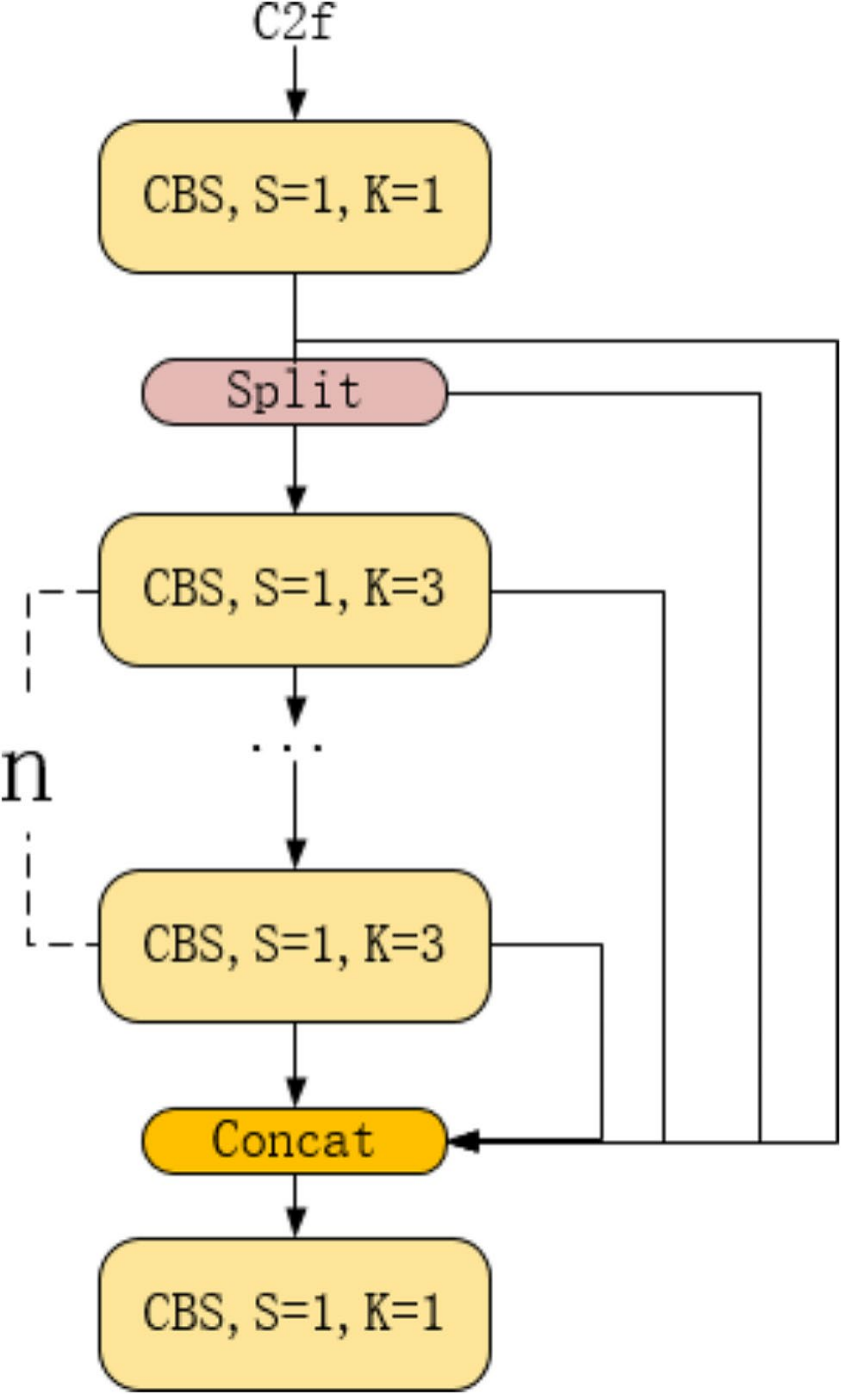
C2f architecture diagram

C2f is a residual module, which is composed of CBS through segmentation, fusion, and other operations, as shown in Fig. 6. Inside the CBS layer, there is a structure as shown in Fig. 5. Split is a feature segmentation layer, mainly used to divide features into two parts: one part of the features remains unchanged, while the other part undergoes processing through several CBS layers. Concat is a feature fusion layer that combines the processed and unprocessed segmented features.

**Fig. 7:**
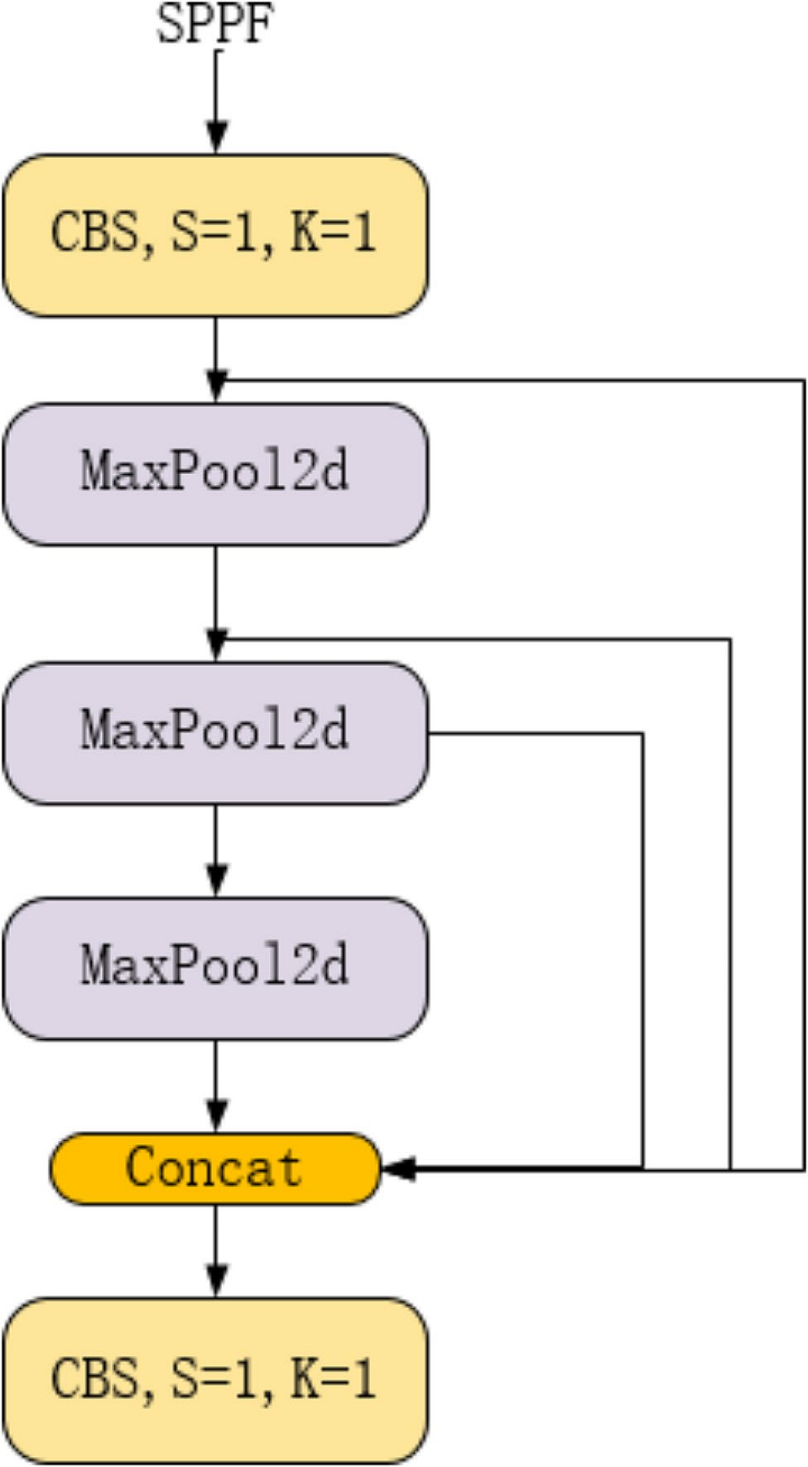
SPPF architecture diagram

The SPPF consists of two parts: the CBS and the pooling layer, as shown in Fig. 7. Inside the CBS layer, there is a structure as shown in Fig. 5. MaxPool2d is a max pooling layer, mainly used to extract prominent features. Concat is a feature fusion layer that combines the features processed by the CBS layer and those extracted by the max pooling layer.

The Head section includes two outputs, one for classification, measured using the BCE binary cross entropy loss function, and one for object recognition, measured using the CIOU + DFL loss function.

The main improvements to the architecture of the model in this article include:

1. A new ROI extraction module for fundus images is added to reduce the image size, allowing the model to focus on smaller areas and speed up training and prediction.
2. By modifying the CIOU loss function, the convergence speed of fundus image training is accelerated, and the accuracy of the result prediction is improved.

### ROI region of interest

**Fig. 8:**
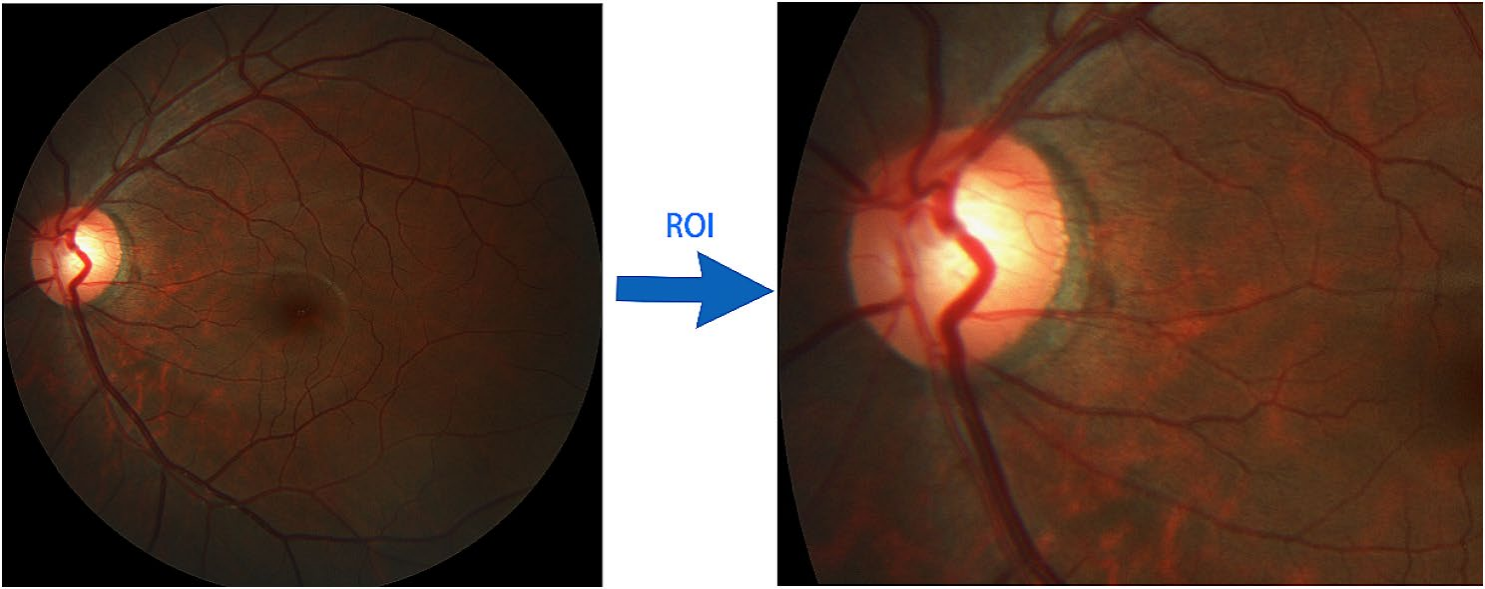
Acquisition of ROI (region of interest)

When most of the optic cup and optic disc segmentation models obtain the ROI of interest, they will additionally establish a neural network model and extract the ROI by training the model. However, this process will consume a lot of resources, and almost half of the training time will be used to extract the ROI. When the final model is segmented, it will also consume a lot of time to obtain the ROI. In order to improve the efficiency of training and prediction, the model in this article uses traditional image cropping techniques to obtain the ROI. By analyzing the fundus images in the REFUGE dataset, a general cropping algorithm is summarized to obtain the ROI. The final algorithm is shown in Formula 1. The height ranges from 0.25 to 0.65, which is based on the data set’s images by first taking height values from 0.1 to 0.8, then checking to see if it can be further reduced, and finally obtaining the height values of 0.25–0.65 through multiple iterations. The width ranges from 0 to 0.5, which is obtained in a similar manner and the processing result of the ROI is shown in Fig. 8.

1 $$\begin{aligned} & {\text{min}}\left( {{\text{height}}} \right)={\text{height*}}0.25 \\ & {\text{max}}\left( {{\text{height}}} \right)={\text{height*}}0.65 \\ & {\text{min}}\left( {{\text{width}}} \right)=0 \\ & {\text{max}}\left( {{\text{width}}} \right)={\text{width*}}0.5 \\ \end{aligned}$$

### Loss function optimization

The regression loss function for the YOLOv8 border is in the form of CIoU + DFL [42], where the CIoU function formula is shown in formula 2.

2 $$\begin{aligned} & {\text{CIoU}}={\text{IoU}} - \left( {\frac{{{p^2}\left( {{\varvec{b}},{{\varvec{b}}^{{\varvec{g}}{\varvec{t}}}}} \right)}}{{{{({w^c})}^2}+{{({h^c})}^2}}}+{\text{av}}} \right) \\ & {\text{v}}=\frac{4}{{{\pi ^2}}}{\left( {arctan\frac{{{w^{gt}}}}{{{h^{gt}}}} - arctan\frac{w}{h}} \right)^3} \\ & {\text{a}}=\frac{v}{{\left( {1 - IoU} \right)+v}} \\ \end{aligned}$$

**Fig. 9:**
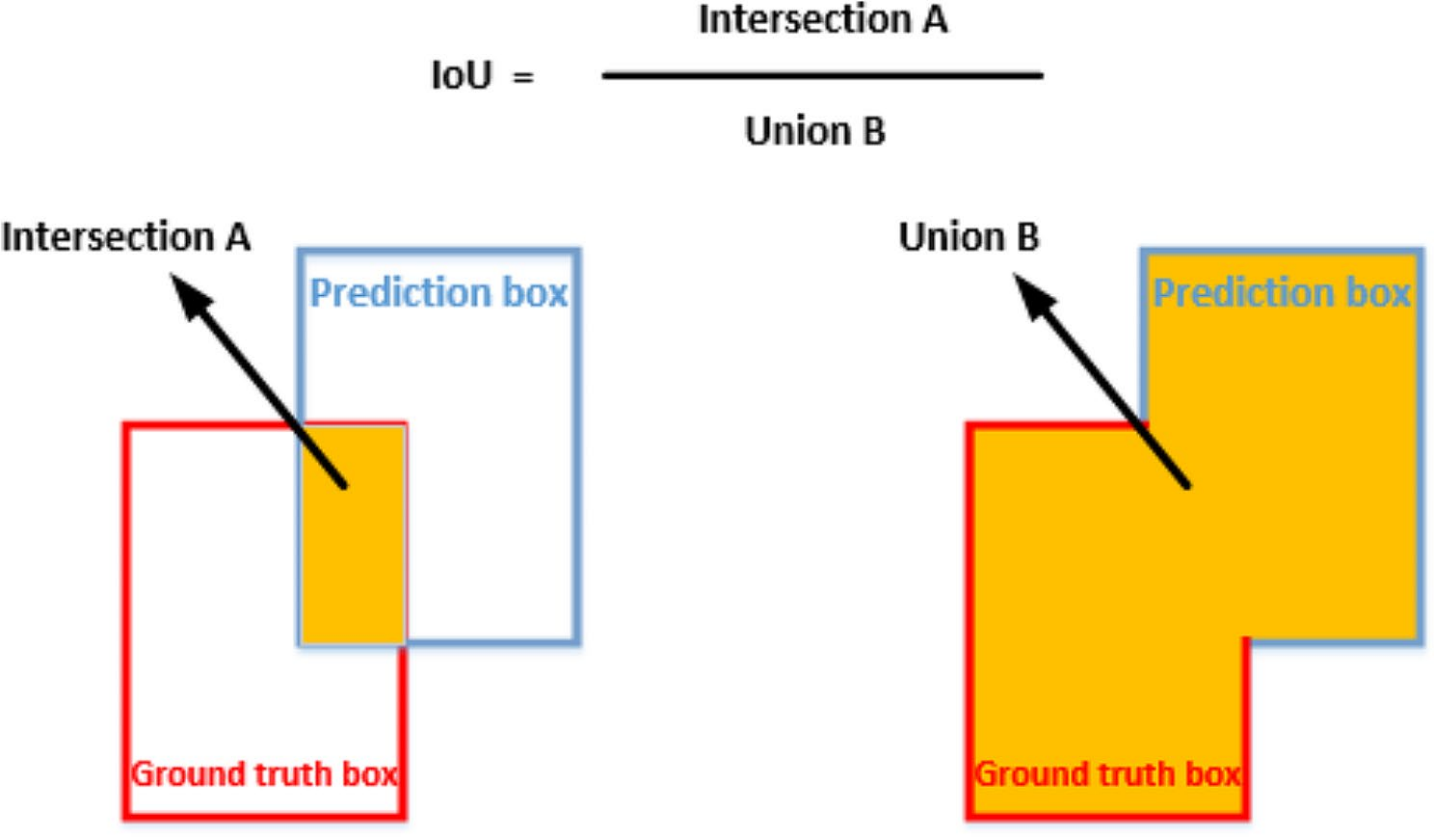
IoU calculation

IoU is the union of the ground truth box and the prediction box divided by the intersection of the ground truth box and the prediction box, as shown in Fig. 9.

**Fig. 10:**
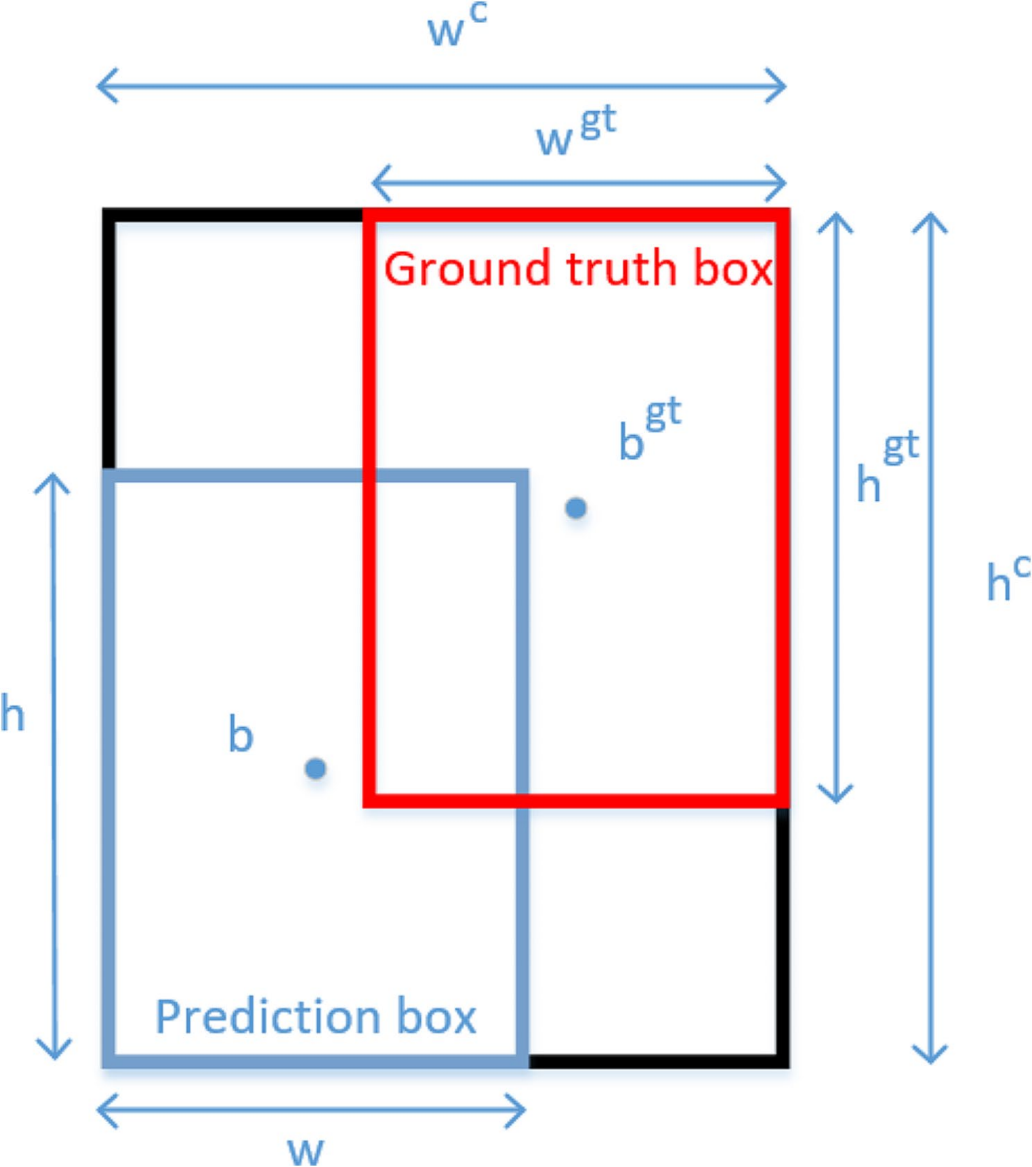
CIoU formula representation

${p}^{2}(b,{b}^{gt})$ is the distance between the center points of the prediction and ground truth boxes, w^c^,h^c^ are the width and height of the minimum bounding rectangle, v is the similarity factor for aspect ratio, and w, h, w^gt^, h^gt^ are the width and height of the prediction and ground truth boxes, respectively, as shown in Fig. 10.

The article [43] found that the following problems exist in CIoU through research:

1. The IoU-based loss function CIoU cannot accurately describe the bounding box, resulting in slow convergence and inaccuracy of the model.
2. The loss function CIoU ignores the imbalance between positive and negative samples, which means that a large number of prediction boxes with small overlap areas with the target box account for the majority of the contribution in the final bounding box optimization.

Based on the above research, article [43] proposed Focal-EIoU, which explicitly measures the differences in three geometric factors in the bounding box, namely the overlap area, center point, and side length. On multiple target detection datasets and models, the Focal-EIoU loss outperforms existing IoU-based losses, and provides greater robustness for small datasets and noisy bounding boxes.

In this paper, the regression loss function of YOLOv8 is replaced from CIoU to Focal-EIoU, as shown in formula 3.

3 $$\eqalign{& {{\rm{L}}_{EIoU}} = {L_{IoU}} + {L_{dis}} + {L_{asp}} = \cr & 1 - IoU + \cr & {{{d^2}\left( {b,{b^{gt}}} \right)} \over {{{\left( {{w^c}} \right)}^2} + {{\left( {{h^c}} \right)}^2}}} + {{{d^2}\left( {w,{w^{gt}}} \right)} \over {{{\left( {{w^c}} \right)}^2}}} + {{{d^2}\left( {h,{h^{gt}}} \right)} \over {{{\left( {{h^c}} \right)}^2}}} \cr & {{\rm{L}}_{Focal - EIoU}} = Io{U^\gamma }{{\rm{L}}_{EIoU}} \cr}$$

${p}^{2}(b,{b}^{gt})$ is the distance between the center points of the prediction and ground truth boxes, ${d}^{2}\left(w,{w}^{gt}\right)$ and ${d}^{2}\left(h,{h}^{gt}\right)$ are the width and height ratios of the prediction and ground truth boxes, respectively, and γ is a hyperparameter used to control the curvature of the curve.

## Results

### Data preparation

The model in this article combines 400 training sets and 200 validation sets from the REFUGE dataset as the training data for the model in this article, with another 200 validation sets as the validation data, and 400 test sets as the test data.

### Experimental equipment

Hardware environment: The training of the model in this article is completed on a desktop computer. Considering the comparison of training time, CPU is used instead of GPU for data training to enlarge the training time. The CPU model is Intel Core i7-9700 with a frequency of 3.0 GHz. The memory size is 32G, and the disk is a 1T solid state drive. Software environment: The operating system used for the experimental work is Windows Server 2019, and the programming language used to build the network model is Python3.9. In order to ensure adequate fitting, the training was iterated 50 times. Due to the memory limitations of the experimental equipment, 600 images were divided into groups of 16 and trained in batches of 600 each. The input of the model is a random size image, and the output is the coordinates of multiple optic disc and optic cup edges.

### Evaluation indicators

The performance of image segmentation can be compared using different algorithm models through evaluation metrics, including precision, recall, P-R curve, F1 score [44], etc. However, these metrics are based on IoU, the intersection-union ratio of the prediction box and the ground truth box. Only after determining the threshold of IoU for detecting the target can these metrics be calculated. As the model predicts the optic cup and optic disc, there are not many noisy boxes. Therefore, this paper sets a relatively loose IoU threshold of 0.5: any predicted box with an intersection over union (IoU) greater than 0.5 with the ground truth box is classified as positive, otherwise it is classified as negative.

There are four types of evaluation indicators: TP, FN, FP, and TN.

- **True Positive (TP)**: The true class of the sample is positive, and the model predicts a positive result, making the prediction correct.
- **True Negative (TN)**: The true class of the sample is negative, and the model predicts it to be negative, making the prediction correct.
- **False Positive (FP)**: The true class of the sample is negative, but the model predicts it to be positive, making the prediction error.
- **False Negative (FN)**: The true class of the sample is positive, but the model predicts it to be negative, making the prediction error.

Precision refers to the ratio of correctly predicted positive samples among all predicted positive samples, and its calculation method is shown in formula 4.

4 $${\text{precision}}={\text{TP}}/\left( {{\text{TP}}+{\text{FP}}} \right)$$

Recall refers to the ratio of correctly predicted positive samples to the total number of true positive samples, and its calculation method is shown in formula 5.

5 $${\text{recall~}}={\text{TP}}/\left( {{\text{TP}}+{\text{FN}}} \right)$$

F1 is the harmonic mean of precision and recall, which can comprehensively consider the accuracy and completeness of the classifier. The maximum is 1 and the minimum is 0. The higher the score, the better the performance of the classifier. Its calculation method is shown in formula 6.

6 $${\text{F}}1=2{\text{*}}\,(\text{p}{\text{recision~*~recall)}}/({\text{precision}}+{\text{~recall)}}$$

### Compare

Because of the limitations of traditional algorithms, the accuracy of traditional model predictions has been restricted. The accuracy of existing deep learning algorithms has already surpassed that of traditional algorithms, and accuracy is a prerequisite for image recognition tasks. Therefore, the algorithms compared in this paper are all deep learning-based [45]. 

Reference [46] compared the performance of various algorithms based on U-Net improvements in the task of segmenting the optic cup and disc. The f1 scores of algorithms such as M-Net, CE-Net, etc., did not show significant improvement over U-Net, and in some cases, M-Net’s f1 score was even lower than that of U-Net. Therefore, this paper overall compares with the U-Net model. Furthermore, algorithms based on U-Net improvements are essentially fully convolutional networks, and their training speed has not fundamentally changed.

**Table 1:** Comparison of model training

Model	Training speed (hour)	Prediction speed for a single image (second)	F1 score
U-Net	213.8	6	0.84
yolov8	55.8	1	0.997
yolov8 + ROI	9.5	0.3	0.997
yolov8 + ROI + Focal-EIOU	8.0	0.3	0.999

**Fig. 11:**
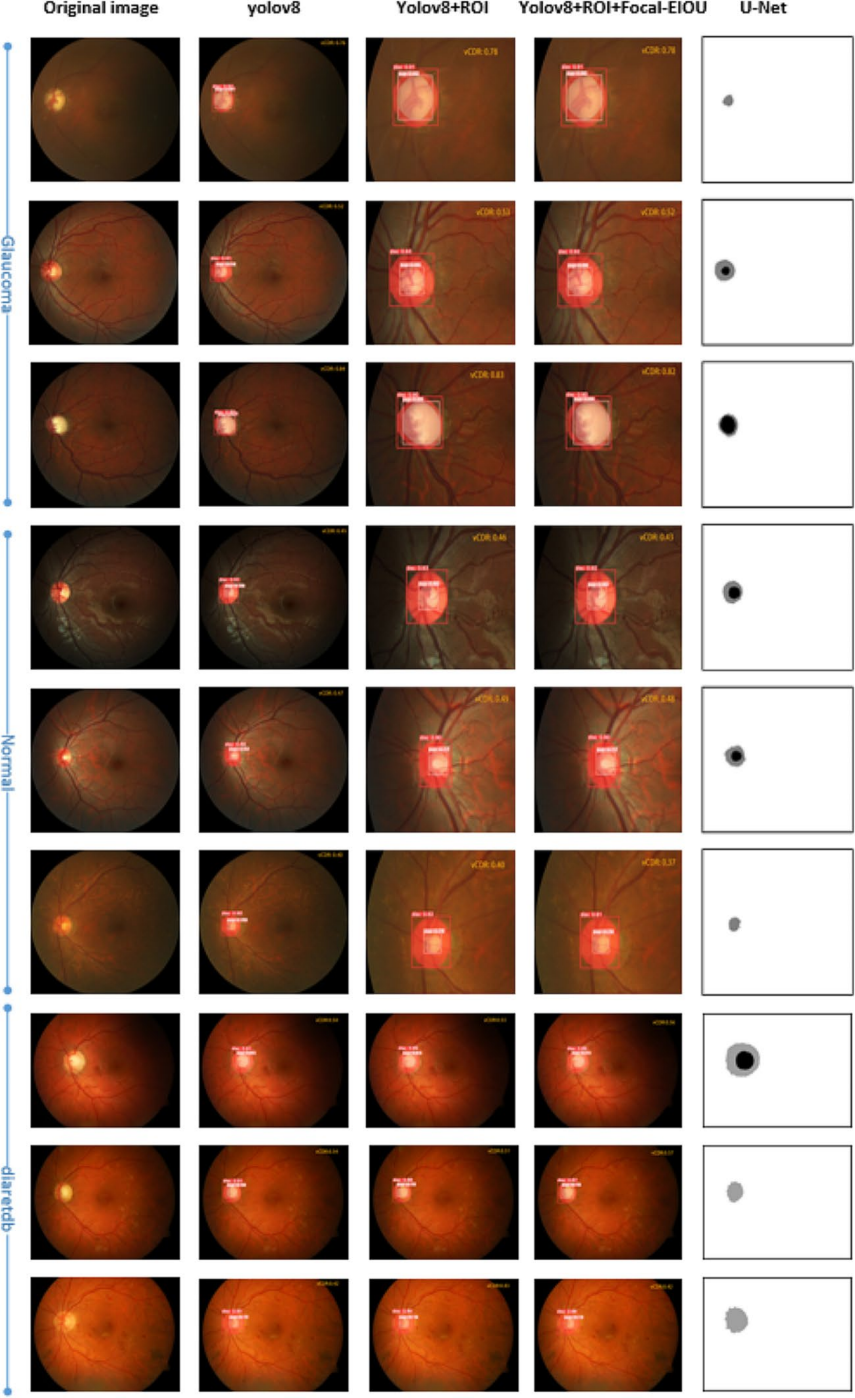
Prediction results of various models

**Fig. 12:**
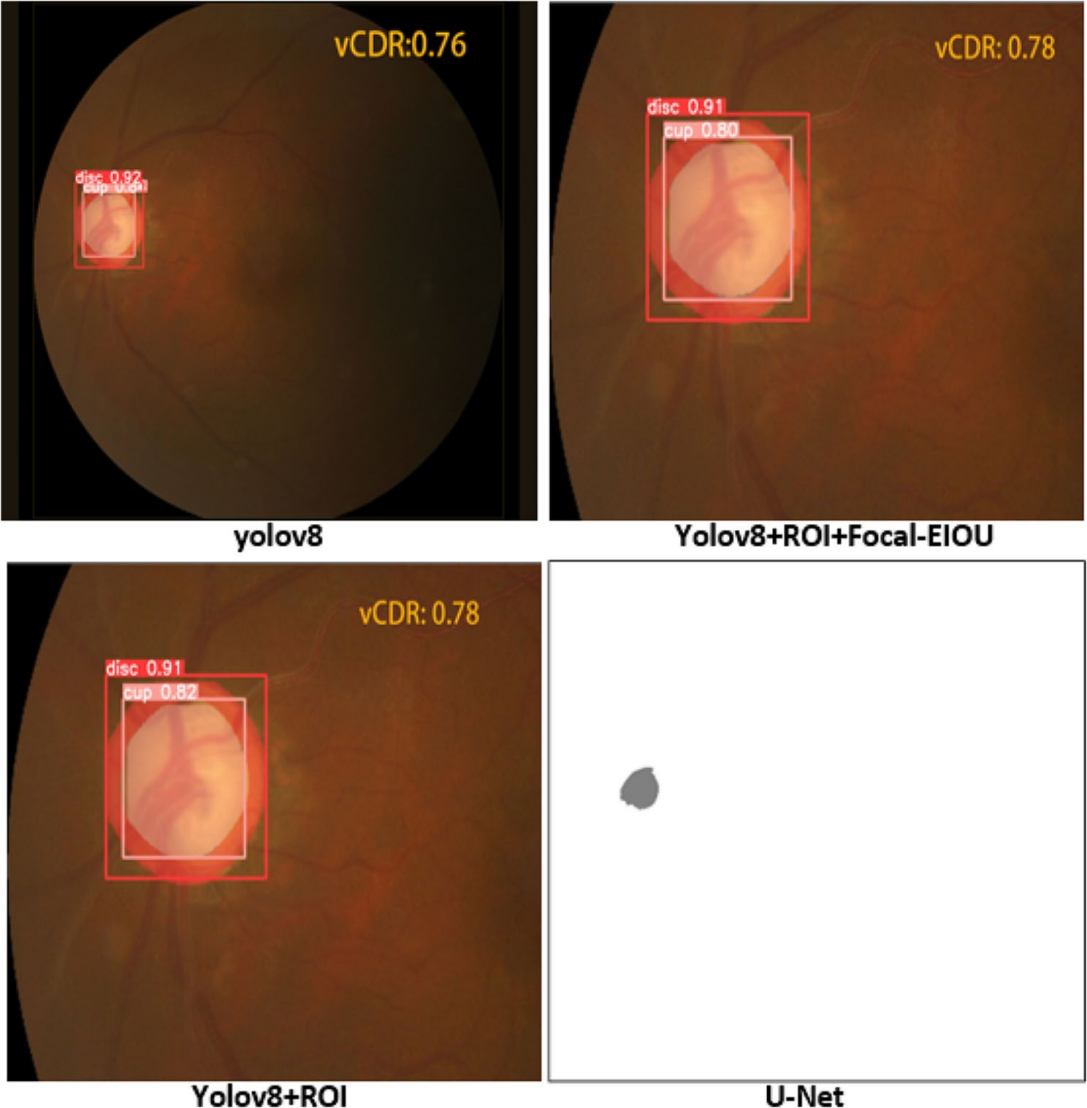
One of the prediction results

From Table 1, it can be found that the algorithm of yolov8 itself is far superior to the U-Net algorithm in terms of speed and segmentation accuracy. The improved ROI module and Focal-IOU loss function in this paper greatly shorten the training time and prediction time of fundus images, which is very important for large-scale image training. The improvement of the Focal-IOU loss function also increases the segmentation accuracy. In addition to taking pictures at random from the REFUGE dataset, this paper also randomly selects three pictures from the public dataset DiaRetDB (DiaRetDB is a public database used for evaluating and assessing algorithms for diabetic retinopathy detection.) for comparison. Figure 11 shows the prediction results of each model, and Fig. 12 shows an enlarged image of the prediction results.

The decimals after disc and cup indicate the confidence of the OD and OC classification results. As can be seen from the figure, the improved algorithm in this article accurately segments the OD and OC of the fundus image, calculates the vCDR, and accurately screens the fundus image for glaucoma based on the vCDR. The U-Net segmentation result has poor accuracy and cannot accurately segment the OD and OC completely.

**Fig. 13:**
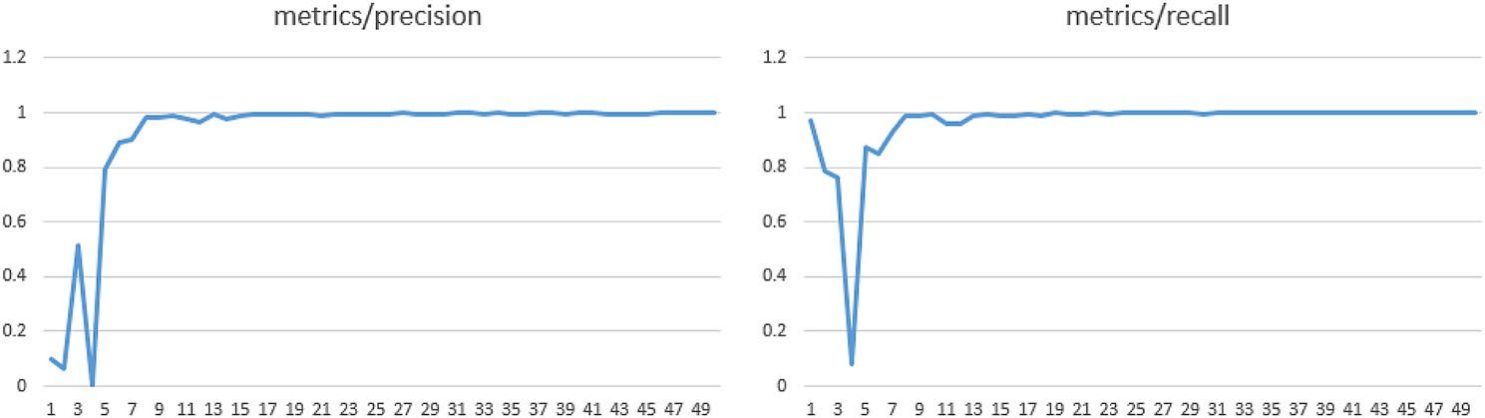
Training metrics

Figure 13 shows the changes in the relevant evaluation metrics (precision and recall) of the model in this article as the number of training iterations increases.

## Conclusion

We applied the improved YOLOv8 algorithm (adding the ROI module and modifying the bounding box regression loss function) to the segmentation task of OD and OC in fundus images. By training the REFUGE dataset, we obtained a model that can calculate the cup-disc ratio of fundus images. This model only needs to input a fundus image, and it will output an image with OD and OC segmentation and the result of cup -disc ratio. In addition, in order to compare the segmentation performance of this model, we also trained the current mainstream U-Net segmentation model using the same dataset. Through comparison, we found that our improved model is far superior to the U-Net model in terms of training speed, segmentation performance, and segmentation accuracy. Based on the above conclusions, in our future work, we will continue to explore the segmentation research of small lesions (microaneurysms, exudates, hemorrhages, etc.) in fundus images based on this improved model.

## Data Availability

No datasets were generated or analysed during the current study.
